# Transcytosis of LDL Across Arterial Endothelium: Mechanisms and Therapeutic Targets

**DOI:** 10.1161/ATVBAHA.124.321549

**Published:** 2025-02-27

**Authors:** Israel O. Bolanle, Gaetan C. de Liedekerke Beaufort, Peter D. Weinberg

**Affiliations:** 1 Department of Bioengineering, Imperial College London, United Kingdom.

**Keywords:** caveolae, cholesterol, coronary disease, permeability

## Abstract

Transport of LDL (low-density lipoprotein) from plasma to arterial intima is thought to be rate limiting in the development of atherosclerosis. Its variation likely determines where lesions develop within arteries and might account for some of the currently unexplained difference in disease susceptibility between individuals. It may also be critical in the development of lipid-rich, unstable plaques. Mechanisms have been controversial but recent evidence suggests that caveolar transcytosis across endothelial cells is the dominant pathway. Receptors involved are LDLR (LDL receptor), SR-B1 (scavenger receptor class B type 1), and ALK1 (activin receptor-like kinase 1). The role of LDLR is influenced by IL-1β (interleukin-1β); the role of SR-B1 by HDL (high-density lipoprotein), DOCK4 (dedicator of cytokinesis 4), GPER (G-protein–coupled estrogen receptor), and HMGB1 (high mobility group box 1); and the role of ALK1 by BMP (bone morphogenetic protein) 9. Additionally, BMP4 stimulates transcytosis and FSTL1 (follistatin-like 1 protein) inhibits it. Fundamental transcytotic mechanisms include caveola formation, undocking, trafficking, and docking; they are influenced by cholesterol-lowering agents, MYDGF (myeloid-derived growth factor), MFSD2a (major facilitator superfamily domain containing 2a) in the blood-brain barrier, and inhibitors of dynamin-2 and tubulin polymerization. The relative merits of different therapeutic approaches are discussed, with statins, colchicine, benzimidazoles, and metformin being existing drugs that might be repurposed and salidroside and glycyrrhizic acid being nutraceuticals worth investigating. Finally, we discuss evidence against the ferry-boat model of transcytosis, the contributions of receptor-mediated, fluid-phase, and active transcytosis, and where inhibition of transcytosis might be most beneficial.

**Highlights**

- Elevated transport of low-density lipoprotein across arterial endothelium is a major risk factor in the development of atherosclerosis, and hence coronary heart disease and stroke, but is not currently a therapeutic target.
- After decades of debate, it now appears that this transport occurs by transcytosis involving caveolae, and receptor-mediated active processes are responsible for some of that transcytosis.
- The identification of the receptors and their coactivators, as well as the discovery of molecular processes involved in transcytosis itself, suggests new therapeutic targets and repurposing of existing drugs with good safety profiles.

A characteristic feature of early atherosclerosis is the accumulation of lipid, particularly cholesterol, within the inner layers of the arterial wall. Evidence that this lipid derives from circulating lipoproteins includes the high risk of cardiovascular disease associated with hyperlipoproteinemia, the beneficial effects of lipid-lowering therapy, the development of atherosclerosis-like disease in animals in which plasma lipoprotein levels have been elevated, and the predilection of atherosclerosis for arterial sites where net uptake of circulating lipoproteins is elevated.^1–4^ LDL (low-density lipoprotein) is the main cholesterol carrier in the human circulation, and the plasma concentration of LDL is a particularly powerful risk factor for human cardiovascular disease.^1,5^

Please see www.ahajournals.org/atvb/atvb-focus for all articles published in this series.

The rate of LDL influx into the arterial intima is determined by the product of 2 variables: the concentration of LDL in plasma, which we assume to be well mixed,^6^ and the permeability of the endothelium to LDL,^5^ which appears not to be saturable. Permeability is used here to denote the ease of entry by diffusive, advective, and vesicular pathways, and reverse transport out of the wall is ignored for the time being. Lowering plasma LDL concentrations is the primary therapeutic strategy used today. Lowering permeability should have a similar benefit. However, this possibility is currently underexplored, most likely because the mechanisms by which LDL crosses endothelium are still under investigation.

Early studies by Vasile et al^7^ used electron microscopy to investigate transport of LDL across the endothelium of rat arteries perfused in situ. Transport was found to occur via the plasmalemmal vesicles now termed caveolae and by a mechanism that was not saturated at physiological LDL concentrations, unaffected by the use of heterologous rather than homologous LDL and relatively insensitive to temperature. In the following decades, focus shifted away from transcytosis and toward transport through intercellular junctions around dividing or dying endothelial cells. (Normal junctions are too narrow for the passage of LDL.) However, Chooi et al^8^ found that such hot spots accounted for only about one-tenth of albumin entry in vivo. Their importance may have been particularly overestimated in vitro because the frequency of mitosis and apoptosis is elevated in the absence of chronic exposure to flow.^9^ The weight of recent evidence, including identification of receptors involved and the key role played by the caveolar protein caveolin-1, favors the view that LDL crosses endothelium by a caveolar route.

Further evidence has come from methods that directly image transendothelial transport. In one method, Armstrong et al^10^ added 1,1′-dioctadecyl-3,3,3′,3′-tetramethylindocarbocyanine iodide (DiI)–labeled LDL to the medium overlying endothelium grown on glass coverslips. The tracer was allowed to bind to the cell surface at 4 °C before unbound LDL was washed away, the temperature increased to 37 °C and total internal reflection fluorescence (TIRF) microscopy used to count small foci of tracer in the bottom ≈100 nm of the cell. The appearance of tracer in this layer does not conclusively demonstrate exocytosis, but that was ameliorated by selecting vesicles that were relatively immobile and disappeared over time. Good evidence for LDL transcytosis was obtained.

A second method was developed from the work of Dubrovskyi et al,^11^ who added fluorescein isothiocyanate–labeled avidin, which is similar in size to albumin, to the medium above an endothelial monolayer cultured on biotinylated gelatin. The tracer traveled across the endothelium and then bound to the underlying biotin; its distribution could be imaged by en face fluorescence microscopy and compared with the overlying structures to determine the pathway by which it crossed. It unequivocally traversed the monolayer through junctions between ≥2 neighboring cells. Ghim et al^12^ increased the size of the tracer by using larger labels. When fluorescein isothiocyanate was replaced with a fluorescent protein, giving a size similar to HDL (high-density lipoprotein), the tracer again crossed through junctions, but only where ≥3 cells met. When a quantum dot was used as the label, giving a size similar to LDL, the tracer instead crossed through cells; there was no transport via intercellular junctions, supporting the view that LDL must also be transported by a transcellular route.

If transcytosis is the major mechanism, that opens new possibilities for reducing transport and, by implication, atherosclerosis. In the following, we review targets that arise from the identification of receptors and pathways and those arising from the fundamental transcytotic machinery.

## Receptors and Pathways as Targets

### LDLR

Dehouck et al^13^ demonstrated a role for the LDLR (LDL receptor). They cocultured bovine brain capillary endothelial cells and astrocytes on opposite sides of a filter insert, a method that induces blood-brain barrier (BBB) properties in the endothelial monolayer, and added LDL to the endothelial side. Tracer crossed the monolayer through a process that was abrogated by a 20-fold excess of unlabeled LDL or by incubation with a monoclonal antibody that blocked the binding domain of the LDLR.

That was surprising since an earlier study by Wiklund et al^14^ had shown no difference in the rate of entry of native and reductively methylated rabbit LDL into rabbit aortic wall in vivo; native LDL is recognized by the LDLR but methylated LDL is not. A likely explanation is that LDLR-mediated transcytosis is confined to the BBB. In vitro studies have found evidence against its occurrence in cultured endothelium derived from adrenal cortex,^13^ human coronary artery,^10^ or human aorta.^15^ However, a recent publication by Jang et al^16^ suggests there may be situations where that is not the case. They used the TIRF microscopy method to show that transcytosis of prebound LDL across human coronary artery endothelial cells was increased by IL-1β (interleukin-1β). The additional transcytosis, but not baseline transcytosis, was mediated by the LDLR (and was independent of caveolin-1). In vivo results were consistent with these in vitro observations: IL-1β increased LDL transcytosis in the aortic arch of wild-type but not *Ldlr*^*−/−*^ mice.

Despite the results of Jang et al,^16^ inhibition of the LDLR seems an improbable method for reducing atherosclerosis, even in situations where IL-1β is elevated, because the LDLR is strongly antiatherogenic: its absence in familial hypercholesterolemic patients, Watanabe heritable hyperlipidemic rabbits, and *Ldlr*^*−/−*^ mice leads to dramatically accelerated disease. Inhibition of the LDLR would need to be arterial and not hepatic.

### SR-B1

Armstrong et al^10^ obtained evidence that SR-B1 (scavenger receptor class B type 1, which they termed SR-BI) plays a role in transcytosis of DiI-labeled LDL across cultured coronary artery endothelium. The evidence included: (1) partial colocalization of DiI-LDL and SR-B1 immunostaining that was abrogated by excess unlabeled LDL, (2) increased transcytosis of DiI-LDL when SR-B1 was overexpressed and decreased transcytosis when it was knocked down, and (3) decreased transcytosis in the presence of excess HDL, the canonical SR-B1 ligand. Furthermore, subendothelial tracer accumulation in the thoracic aortas of SR-B1 gene knockout (*Scarb1*^*−/−*^) mice was decreased compared to wild-type mice after perfusion ex vivo with a solution containing LDL.

Huang et al^15^ confirmed and extended the in vitro work on SR-B1, provided evidence for a significant role of SR-B1 in vivo, and established underlying mechanisms. In cultured monolayers of human aortic endothelial cells, LDL transcytosis was decreased by SR-B1 knockdown, by mutating SR-B1 so that it could not bind LDL, by a blocking antibody, and by the SR-B1 inhibitor BLT-1 (block lipid transport-1). Entry of LDL into the mouse aorta in vivo was reduced by endothelial-specific *Scarb1*^*−/−*^ knockout or by an SR-B1 blocking antibody. Endothelial knockout of SR-B1 on an *Apoe*^*−/−*^ background approximately halved atherosclerosis compared with control *Apoe*^*−/−*^ mice.

Huang et al^15^ went on to show that SR-B1 requires a partner protein, DOCK4 (dedicator of cytokinesis 4), to facilitate LDL transcytosis. Knockdown of DOCK4 suppressed SR-B1–dependent LDL transcytosis in cultured cells, and both *Scarb1* and *DOCK4* mRNA levels were increased in the atherosclerosis-prone lesser curvature of mouse and human aortic arch. The combination of LDL, SR-B1, and DOCK4 caused Rac1 (Ras-related C3 botulinum toxin substrate 1) activation, and preventing this activation reduced LDL uptake in culture.

A number of physiological pathways regulate the role of SR-B1. Consistent with the well-established antiatherogenic effect of estrogen, Ghaffari et al^17^ found that transcytosis of LDL was higher in human coronary artery endothelial cells from males and a postmenopausal female than in cells from younger females. In male but not younger female cells, transcytosis was reduced ≈50% by adding estrogen to the medium. Estrogen downregulated *SR-B1*, and after *SR-B1* knockdown in male cells, there was no additional reduction of transcytosis by estrogen. Effects of estrogen were mediated by the GPER (G-protein–coupled estrogen receptor) but not estrogen receptors α and β. GPER exerted its effect via the EGFR (epidermal growth factor receptor). Importantly, the effects of estrogen on *Scarb1* mRNA were seen in male coronary endothelial cells but not in hepatic cells; that may be because in the coronary endothelium but not hepatic cells, *GPER* mRNA predominated over mRNA for estrogen receptors α and β. An unresolved issue is the apparent discrepancy with an earlier study^18^ that failed to show an effect of estrogen on aortic LDL permeability in ovariectomized rabbits in vivo.

Subsequent work by Ghaffari et al^19^ identified a further pathway regulating SR-B1–mediated transcytosis of LDL. Knockdown of HMGB1 (high mobility group box 1), an abundant nuclear, cytoplasmic, and secreted protein, reduced transcytosis of LDL in human coronary artery endothelial cells by around 40% without affecting albumin transcytosis. The knockdown reduced SR-B1 expression, and knockdown of both SR-B1 and HMGB1 had no additional effect on LDL transcytosis over the single knockdowns, indicating a common pathway. Similarly, knockdown of either HMGB1 or the transcription factor SREBP2 (sterol regulatory element-binding protein 2) reduced transcytosis, but knockdown of both had no additional effect. The influence of HMGB1 appeared to be mediated by alteration of the half-life of SREBP2, and secreted HMGB1 was not involved. Consistent with these experiments on cultured endothelium, accumulation of labeled LDL in the mouse aortic arch was reduced 50% by an endothelial cell–specific knockout of *Hmgb1*, whether on a wild-type or *Ldlr*^*−/−*^ background, and atherosclerosis was reduced in the latter compared with the *Ldlr*^*−/−*^ single knockout.

In summary, endothelial SR-B1 and DOCK4 facilitate LDL transcytosis, possibly via Rac1 activation. SR-B1–mediated LDL transcytosis is blocked by BLT-1 and likely by less toxic inhibitors.^20,21^ It is also decreased by HDL and by estrogen acting via GPER and EGFR. It is increased by HMGB1 via SREBP2.

Proposing SR-B1 as a therapeutic target suffers from the same problem as proposing the LDLR: hepatic SR-B1 is involved in reverse cholesterol transport. Thus, nonspecific knockdown of SR-B1 in *Apoe*^*−/−*^ mice leads to greatly accelerated atherosclerosis,^22^ as does selective silencing of SR-B1 in hepatocytes,^15^ the latter observation presumably explaining the former. It cannot be assumed that inhibition of SR-B1 will have a beneficial effect on human atherosclerosis. (The effect on coronary disease of rare mutations in the *SCARB1* gene is under debate.^23,24^) Estrogen is already known to be atheroprotective, but effects on SR-B1–mediated transcytosis add a new mechanism; G-1 is a specific agonist for GPER,^25^ through which estrogen affects SR-B1, and that might confer an advantageous, greater effect in arterial endothelial cells than in hepatic cells. HDL is also already known to be atheroprotective. apoA-II^26^ and nanoparticle mimetics of HDL^27^ are effective in cancers in which SR-B1 is upregulated, but they presumably compete with HDL involved in reverse cholesterol transport. BLT-1 was developed to inhibit SR-B1–mediated transfer of cholesterol and cholesteryl esters between HDL and cells and so is also likely to have an adverse effect on reverse cholesterol transport. The role of DOCK4 in SR-B1–mediated transport of HDL is currently a matter of debate.^15,28^ HMGB1 has been inhibited in vivo with glycyrrhizic acid, which thereby reduced asthma induced by toluene diisocyanate in mice.^29^ Investigations of the effect of glycyrrhizic acid and of metformin, another HMGB1 inhibitor, on LDL transcytosis and disease appear warranted because the latter is beneficial overall in type 2 diabetes.

### ALK1

Kraehling et al^30^ used a genome-wide screen to establish that a TGF-β (transforming growth factor-β) type 1 receptor, ALK1 (activin receptor-like kinase 1; or ACVRL1 [activin A receptor like type 1]), is also involved in endothelial transcytosis of LDL. Direct binding of LDL and ALK1 was demonstrated by surface plasmon resonance, although the affinity was lower than for the LDLR. TIRF microscopy showed that transcytosis of prebound LDL across human coronary artery endothelial cells was reduced by ≈50% after silencing of *ACVRL1*, and ALK1 overexpression increased transcytosis. Transendothelial transport of LDL through coronary artery endothelium grown on Transwell filters was also reduced by around 50% after silencing of *ACVRL1*.

BMPs (bone morphogenetic proteins) 9 and 10 are growth factors of the TGF-β superfamily with high affinity for ALK1.^31^ A subsequent article^32^ showed that BMP9 and, to a lesser extent, BMP10 triggered endocytosis of ALK1. Pretreatment of human coronary artery endothelium with BMP9 to internalize ALK1 nearly halved transcytosis of prebound DiI-LDL, assessed by the TIRF method. The reduction was similar to that caused by silencing *ACVRL1*.

The important role of ALK1 seen in culture has recently been confirmed in vivo.^33^ Deletion of *Acvrl1* in arterial endothelial cells of mice was achieved without loss of viability. When LDLR was also suppressed, using PCSK9 (proprotein convertase subtilisin/kexin type), and mice were placed on a Western diet, *Acvrl1* deletion reduced lipid deposition by 50% in the aortic root and by more in the brachiocephalic artery. Similar results were obtained when an *Ldlr*^*−/−*^ strain was used instead of PCSK9.^33^

Treatment of human coronary artery endothelial cells with an antibody that blocked binding of LDL to ALK1 but did not influence BMP9 or BMP10 signaling gave a 50% reduction in transcytosis of prebound LDL.^33^ In *Ldlr*^−*/*−^ mice, the antibody reduced lesion area, lipid deposition, and apoB accumulation by around 50%, and it also doubled the rate of lesion regression.^33^

ALK1 inhibitors are under development as anticancer drugs because they suppress angiogenesis. We are not aware of any studies examining their ability to prevent LDL transcytosis. The monoclonal antibody developed by Lee et al^33^ blocks LDL binding while leaving BMP9 and BMP10 signaling intact. Interventions to reduce atherosclerosis by interfering with LDL transcytosis would have to be given chronically, so a monoclonal would not be economically viable, but it may assist in the identification of the apoB100-binding site, which could allow the development of other inhibitors.

BMP9, which reduced ALK1-mediated LDL transcytosis, has been used therapeutically in a mouse model of pulmonary arterial hypertension^34^; it was administered at 75 ng IP daily for 4 weeks. As well as reversing pulmonary arterial hypertension, BMP9 inhibited lipopolysaccharide-induced Evans blue dye extravasation in the mouse lung, although the route of extravasation was not determined.

### FSTL1 and BMP4

Ghim et al^35^ showed that transcytosis of LDL was inhibited by FSTL1 (follistatin-like 1 protein; also known as TGF-β–induced clone 36 or follistatin-related protein). The hypothesis arose from experiments that applied an LDL-sized tracer to endothelial cells exposed to different flows. When endothelial cells in dishes or multiwell plates are swirled on an orbital shaker, they experience a relatively low magnitude, highly multidirectional shear stress in the central region, and higher magnitude, more uniaxial shear stress towards the edge^36^; these are putatively proatherogenic and antiatherogenic, respectively.^37^ Paracellular transport of albumin- and HDL-sized tracers was increased in the center and decreased at the edge, compared with static conditions, but transport of the LDL-sized tracer, which occurs through the cells, was depressed uniformly across the well, consistent with a soluble mediator being released in one region, mixing in the swirled medium and reducing transcytosis everywhere.^12^

The presence of a mediator was demonstrated by growing cells only in the center or only at the edge of the swirled well, or under static conditions, and applying the resulting conditioned medium to target cells. Medium conditioned by cells grown under putatively antiatherogenic shear was found to suppress transcytosis across target cells, whereas medium condition by cells grown under putatively proatherogenic shear or under static conditions was ineffective.^35^ Unbiased proteomic analysis and other assays identified the active component as FSTL1, and exogenous recombinant FSTL1 was found to reduce transcytosis of the LDL-sized tracer and of unlabeled LDL applied at physiological concentration.^35^

A further observation was that BMP4 increased transcytosis in culture.^34^ That is of interest because there is higher expression of BMP4 in atheroprone regions of the arterial tree than in protected regions.^38,39^ BMP4 upregulates ALK1,^40^ but it is not known whether that is how it increased transcytosis. The elevated transcytosis produced by BMP4 was abolished by the BMP inhibitor noggin, whereas it was not only abolished but brought below baseline by FSTL1. That is consistent with FSTL1 abolishing the effect of BMP4 and at least one other driver of transcytosis. In that context, the observations that FSTL1 can bind ALK1^41^ and DIP2A (disco-interacting protein 2 homolog A)^42^ may be significant. No interactions of FSTL1 with SR-B1 have been reported.

FSTL1 shares domains with follistatin, which is a known inhibitor of the TGF-β superfamily, as well as ALK1 and DIP2A; it can bind to activin, TGF-β, BMP2 and BMP4, and their receptors.^42,43^ A potential issue for its therapeutic use is that it may have proinflammatory effects,^44^ as well as anti-inflammatory ones.^35^

There are many other BMP4 inhibitors, but, like FSTL1, they are not specific. Noggin inhibits not only BMP4 but also BMPs 2, 5, 7, 13, and 14.^45^ Dorsomorphin, which was the first small molecule developed to inhibit BMP4, and its derivatives LDN-193189, dorsomorphin homolog 1, and K02288 are inhibitors of BMP receptors, such as the BMP receptor type 1 ALK2, but affect other signaling pathways as well.^45^ It is unclear how much the lack of specificity matters. Noggin, for example, is under investigation as a therapeutic agent in spinal cord injury.^46^ (Interestingly, K02288 inhibits ALK1.^44^) The development of llama antibodies with highly specific binding to BMP4^45^ may have therapeutic potential.

## Vesicular and Transcytotic Machinery as Targets

The resurgence of interest in the atherogenic role of LDL transcytosis makes it likely that the underlying cellular and molecular mechanisms will become a focus for future research. Currently, however, there is a paucity of definite information,^47,48^ particularly concerning exocytosis, and in this section we consequently examine only targets that have received significant attention to date.

### Vesicle Formation

Here, we focus on the roles of caveolin-1 and cholesterol. Segments of aorta incubated in vitro with LDL took up approximately half as much if they had been obtained from *Cav1*^*−/−*^ mice rather than control mice.^49^ In vivo uptake of LDL was reduced in aortas of mice with an endothelial-specific *Cav1*^*−/−*^ knockout compared with control mice and returned to normal if endothelial-specific caveolin-1 expression was rescued.^50^ A similar effect was seen when *Cav1*^*−/−*^*eNOS*^*−/−*^*Ldlr*^*−/−*^ triple knockout mice were compared with the *eNOS*^*−/−*^*Ldlr*^*−/−*^ double knockout mice^51^. (eNOS [endothelial NO synthase] was knocked out so that the effects of caveolin-1 were independent of its inhibitory influence on the enzyme.) The latter study also showed less atherosclerosis in the triple than in the double knockout. Caveolin-1 deletion likely had an effect on LDL transport simply because caveolae cannot form without it.

A dependence on membrane cholesterol has also been observed: when cholesterol is reduced, so are the number of caveolae. Thus, Rothberg et al^52^ found that reducing cholesterol content by around 50% did not affect cell viability but reduced the number of caveolae 10-fold. Subsequently, Schnitzer et al^53^ found that the cholesterol-binding antibiotic filipin produced a 50% decrease in the transport of ^125^I-labeled albumin across monolayers of bovine lung microvascular endothelium grown on Transwell filters, while the transport of a smaller tracer was unaffected. The fraction of albumin transport that involved binding of albumin to its cell surface receptor gp60 (albondin) was 50%. It thus seems likely that the receptor-mediated transport was abrogated by filipin, whereas another route—probably the paracellular one—was not. A similar result was obtained by cooling, and filipin produced no further effect when the cells were cooled.

Schnitzer et al^53^ obtained a similar result in the perfused rat lung. Rosengren et al^54^ obtained a negative result, but measurement was by a less direct method, and work by Sun et al^55^ supported the conclusions of Schnitzer et al: transport of oxidized LDL across human umbilical vein endothelial cell (HUVEC) monolayers was inhibited >70% by filipin. Uptake and efflux by the cells were also decreased. The observation that 24-hour pretreatment of HUVEC monolayers with LDL increased endocytosis, exocytosis, and transendothelial transport of 70-kDa dextran^56^ may similarly be related to an increase in membrane cholesterol.

On balance, these studies show that inhibition of caveolin-1 or reduction in cholesterol might reduce endothelial permeability to LDL and, thereby, atherosclerosis. However, caveolae have many roles so that there must be a strong preference for repurposing existing drugs that are already known to have a favorable safety profile. We propose that statins could be used for the purpose.

Studies of the effects of statins on caveolin-1 have been motivated mainly to explain their beneficial influence on NO synthesis. Feron et al^57^ found that even 10 nmol/L atorvastatin decreased caveolin-1 protein expression by 75% in bovine aortic endothelial cells in vitro. The effect of the statin was reduced by supplying exogenous cholesterol in the form of LDL. (LDL increased caveolin-1 expression in the absence of atorvastatin as well.) The effect was also reduced by adding mevalonate (the product of HMG-CoA [3-hydroxy-3-methylglutaryl coenzyme A] reductase). Hence atorvastatin appears to decrease caveolin-1 expression by decreasing the products of HMG-CoA reductase synthesized by the endothelial cells themselves, including but perhaps not exclusively cholesterol. The effects of cholesterol have been attributed to the sterol response elements in the promotor of *CAV1*^57^ but may simply reflect interference with the formation of lipid rafts or modification of membrane biophysics. Since caveolin-1 expression was increased in a dose-dependent fashion by the addition of LDL, it is possible that the LDL-lowering effect of statins would further reduce caveolin-1 in vivo, in addition to any direct effects on the endothelial cells themselves.

Concerning evidence that transcytosis is affected, it may be relevant that van Nieuw Amerongen et al^58^ found simvastatin reduced transport of LDL through cultured HUVEC monolayers after permeability had been raised by thrombin. The authors interpreted that as an effect of the statin on intercellular junctions; thrombin is routinely used to widen junctions in vitro.^59^ However, numerous studies cited above show that LDL is transported across monolayers by a transcellular rather than a paracellular route (albeit without thrombin stimulation); exposure to thrombin increases caveolin-1 expression in human lung microvascular endothelial cells and appears also to increase transcytosis, at least of albumin.^60^ Further study with one of the direct visualization techniques described above would be valuable.

Could vesicle formation be reduced by other cholesterol-lowering agents? Water-soluble forms of cyclodextrin, such as methyl-β-cyclodextrin, are widely used to deplete plasma membranes of cholesterol and also reduce caveolin-1 in cultured endothelial cells.^61^ 2-hydroxypropyl-β-cyclodextrin promotes regression of atherosclerosis in mice and is safe to use in human subjects.^62^

Another possibility arises from the observation of Bai et al^63^ that 0.1 μmol/L salidroside decreased caveolin-1 expression in HUVECs by ≈50%, putatively by enhancing its autophagic degradation. Salidroside is a phenylpropanoid glycoside extracted from *Rhodiola* genus; it has a long history of use, but its pharmacological actions have been only partly characterized.^64^

Caveolin-1 (and cavin) is also reduced by MYDGF (myeloid-derived growth factor): *Ldlr*^*−/−*^ mice with an additional monocyte/macrophage-specific *Mydgf* knockout had more LDL transcytosis, arterial LDL uptake, and atherosclerosis than control *Ldlr*^*−/−*^ mice, and the effects could be reversed by bone marrow transplantation or MYDFG overexpression.^65^

### Vesicle Undocking

Here, we consider the GTPase dynamin-2 (the predominant endothelial isoform) and its Src-mediated phosphorylation. Shajahan et al^66^ described how these 2 components of the molecular machinery operate in transcytosis of albumin across rat lung microvessel endothelium. Binding of albumin to endothelial gp60 in culture triggered phosphorylation of Src, Src-mediated phosphorylation of dynamin-2, dynamin-2 oligomerization, dyanmin-2 interaction with caveolin-1, and hydrolysis of GTP; these steps were necessary for scission of caveolae. Dynamin forms a helical collar around the neck of vesicles and, when phosphorylated, pinches the neck, separating it from the plasma membrane. (Caveolin-1 is also a substrate for the Src kinases; its phosphorylation at tyrosine 14, which again can be stimulated by albumin, leads to detachment of caveolae from the plasma membrane, but the resulting free caveolae appear to be larger than normal.^67,68^) It is unclear what happens in fluid-phase transcytosis (ie, transcytosis in which extracellular fluid is imbibed by vesicles before their excision, without binding of specific solutes within it).

A number of dynamin inhibitors have been developed. Dynasore,^69^ a small molecule that rapidly and reversibly inhibits dynamin-dependent endocytosis, is now widely used. There is convincing evidence for off-target effects, but they might also beneficially reduce transcytosis. Thus Park et al^70^ found that dynasore and the structurally similar Dyngo-4a both inhibited fluid-phase endocytosis in fibroblasts, even though these 2 processes remained intact when dynamin 1, 2, and 3 were all knocked out. Furthermore, dynasore reduced the amount of cholesterol in plasma membranes in a dynamin-independent manner.^71^

Armstrong et al^10^ used the TIRF microscopy method to show that 30 µmol/L Dyngo-4a almost eliminated transcytosis of prebound LDL across human coronary artery endothelial monolayers. Ghim et al^35^ found that dynasore more than halved transcytosis of quantum dot–labeled streptavidin tracer across human aortic endothelial monolayers; such transport is presumed to represent fluid-phase or absorptive transcytosis because the existence of receptors recognizing quantum dots or streptavidin is unlikely.

Both inhibitors have been used in vivo, dynasore in studies of rat spinal injury,^72^ mouse osteosarcoma,^73^ and mouse *Shigella* infection,^74^ and Dyngo-4a in studies of botulism in mice and rats,^75^ rotavirus infection in mice,^76^ and sperm viability in mice.^77^ Nevertheless, translation to human subjects in the near term seems unlikely.

### Vesicle Trafficking

Two models for vesicle trafficking in transcytosis are, first, a passive, stochastic process akin to Brownian motion or superdiffusive Brownian motion^78,79^ and, second, that vesicles are actively directed by being tethered to elements of the cytoskeleton,^80^ most likely microtubules; direct evidence for the latter derives from epithelial rather than endothelial cells.^47^ The fact that vesicles occupy up to 35% of the non-nuclear volume of endothelial cells^81^ complicates either mechanism; that figure should be compared with the maximum value (74%) obtainable for close-packed spheres.

The second mechanism would allow for pharmaceutical intervention. As with the inhibition of caveolin-1, microtubules obviously have important physiological roles so the repurposing of existing drugs with favorable safety profiles would be preferred.

Colchicine, an alkaloid derived from *Colchicium autumnale*, has been used for many years to treat gout and other conditions.^82^ In recent years, a number of trials have shown its benefit in cardiovascular disease,^82^ and a bigger role in prevention and management of such disease has been advocated due to its anti-inflammatory actions.^83,84^ Colchicine binds tubulin and inhibits its polymerization. It decreased transport of labeled albumin across cultured bovine aortic endothelial cells, which was dominantly receptor mediated, by ≈80%.^85^ There are numerous reports consistent with colchicine reducing transcytosis in other cell types too; however, transport of LDL does not appear to have been examined.

Benzimidazoles are used for the eradication of intestinal helminths in veterinary and human patients. They act by disrupting microtubule polymerization in the parasites. They attach to the colchicine-binding site on tubulin with different affinities,^86^ and their systemic efficacy, if given orally, would also depend on their absorption by the gut, which is generally poor. Sun et al^55^ found that nocodazole caused an 80% reduction in the transport of oxidized LDL across HUVEC monolayers. Furthermore, it caused an ≈70% reduction in the exocytosis of oxidized LDL that had been preloaded into the cells, giving confidence that transport across the monolayers occurred by transcytosis rather than via a paracellular route.

These compounds are under investigation as cancer-preventing agents because of the role of microtubules in cell division. There are numerous other chemotherapy agents that operate by preventing tubulin polymerization; they could be added to the list of candidates for reducing transcytosis.

### Vesicle Docking

Vesicle docking involves the interaction of N-ethylmaleimide (NEM)–sensitive fusion protein (NSF [NEM-sensitive factor]) with SNAPs (soluble NSF attachment proteins) and SNAREs (SNAP receptors); there is also recruitment of Rab (Ras-associated binding) proteins.^87^ As the name of the fusion protein suggests, this process is inhibited by NEM. Schnitzer et al^88^ showed that NEM halved the transendothelial transport of albumin and tissue accumulation of albumin in rat lungs.

If also applicable to LDL, this would suggest that interfering with vesicle docking could reduce entry into the arterial wall. However, NEM is a general thioalkylating agent.^89^ It affects multiple nontranscytotic processes mediated by vesicles,^87^ and its actions are not restricted to docking; for example, it interferes with the microtubule motors kinesin, Ncd (nonclaret disjunctional), and dynein.^90^ Controversially, Rippe and colleagues found that NEM increased extravasation of albumin^89^ and LDL.^54^ Thus, if attempts are made to interfere with docking, more selective agents would be required.

## Discussion

LDL transport across arterial endothelium is thought to be the rate-limiting step in the initiation of atherosclerosis^91^ and, it has been suggested, in the progression of stable lesions to lipid-rich unstable ones as well.^92^ It likely determines the predilection of atherosclerosis for specific sites within the arterial system, and different rates in different people could underlie part of the unexplained interindividual risk for cardiovascular disease. However, it is underexplored as a therapeutic target. Much recent evidence supports the view that transcytosis is the dominant pathway. Here, we have described the receptors and pathways involved and also outlined general vesicular and transcytotic machinery, with a focus on where antiatherogenic intervention might be practicable. A summary of the mechanisms is given in the Figure, and the targets arising from each mechanism are listed in the Table.

**Table.**
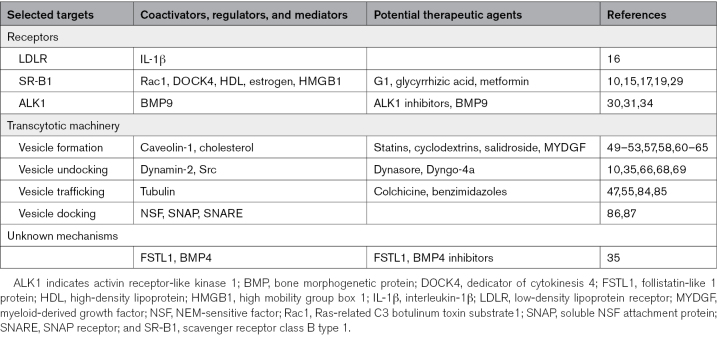
Selected Targets for Therapeutic Modification of LDL Transcytosis Across Endothelium and Related Agents With Potential Therapeutic Utility

**Figure.**
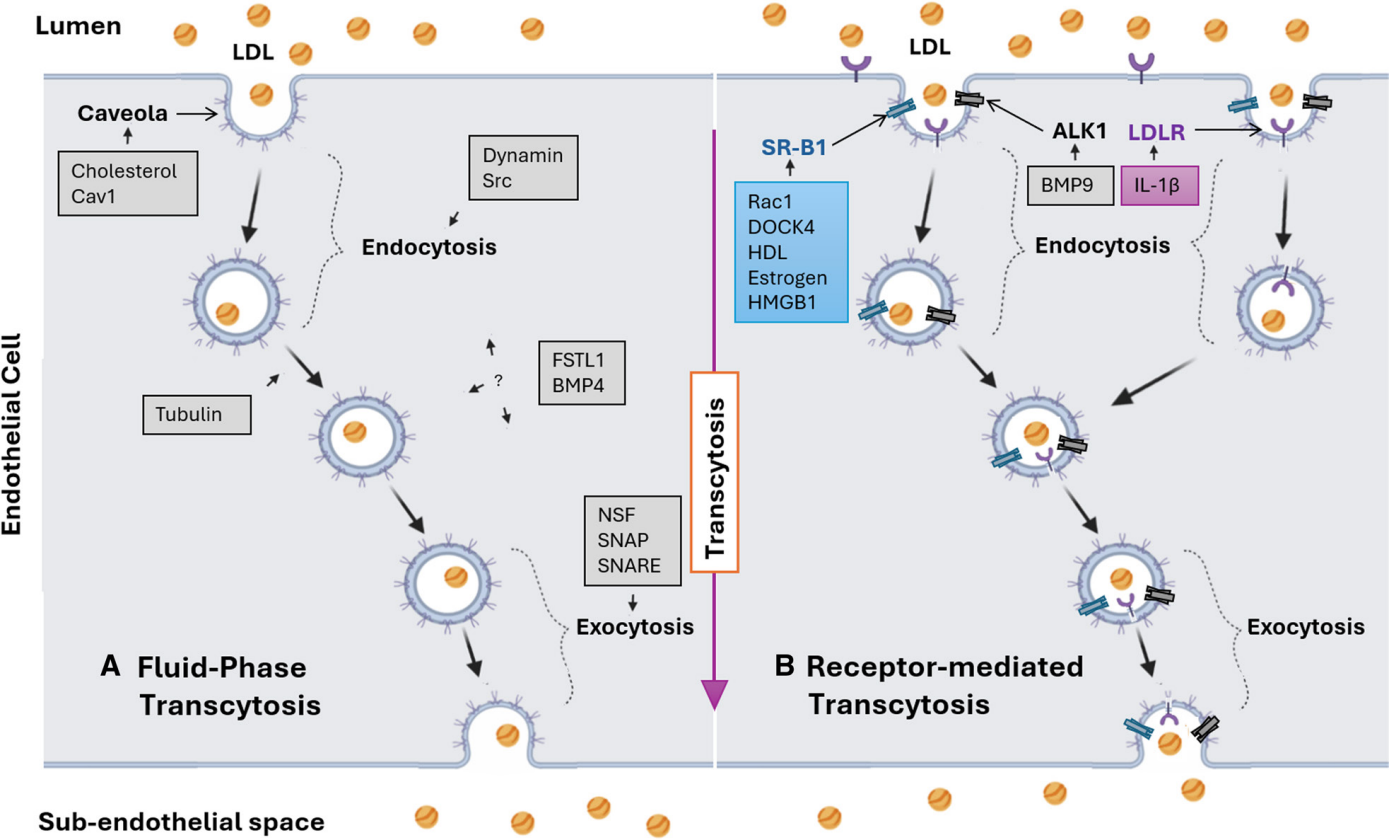
**A schematic showing transcytosis of LDL (low-density lipoprotein) from the vessel lumen to the subendothelium in caveolar vesicles. A**, Fluid-phase transcytosis. **B**, Receptor-mediated transcytosis. In the latter, LDL binds to specific receptors such as the LDLR (LDL receptor), ALK1 (activin receptor-like kinase 1), or SR-B1 (scavenger receptor B type 1). Coactivators and the molecules underlying basic transcytotic machinery are shown in boxes. Cav1, caveolin-1; BMP, bone morphogenetic protein; DOCK4, dedicator of cytokinesis 4; FSTL1, follistatin-like 1 protein; HDL, high-density lipoprotein; HMGB1, high mobility group box 1; IL-1β, interleukin-1β; NSF, NEM-sensitive factor; Rac1, Ras-related C3 botulinum toxin substrate1; SNAP, soluble NSF attachment protein; and SNARE, SNAP receptor.

Not all pathways could be included. For example, transcytosis of LDL in cultured endothelium and ex vivo venous rings was increased by TNF-α (tumor necrosis factor-α),^93^ which opens up the possibility that pharmaceutical or nutraceutical inhibition of TNF-α might be useful. We also did not include pathways that reduce transcytosis in specialized blood-tissue barriers, but their relevance in arteries prone to atherosclerosis may deserve further study. MFSD2a (major facilitator superfamily domain containing 2a) is expressed in endothelial cells of the BBB,^94^ blood-retinal barrier,^95^ and blood-labyrinth barrier^96^ and is responsible for the low rate of transcytosis there; it appears to suppress caveola formation by altering the lipid composition of the plasma membrane^94^ and therefore resembles to some extent the cholesterol-lowering agents described above. Expression of MFSD2a in endothelium of the BBB is under control of surrounding cells. Increases in transcytosis in the BBB are seen after brain injury; they correlate with a decrease in MFSD2A expression and can be reversed by angiopoietin-1,^97^ the sphingosine-1-phosphate receptor 1 agonist CYM-5442,^98^ and GGF2 (glial growth factor 2).^99^ It is not known whether angiopoietin-1, CYM-5442, and GGF2 upregulate MFSD2A expression and decrease transcytosis outside the BBB.

Of the potential therapeutic agents detailed above, a few are discussed further here. MYDGF, FSTL1, and BMP9 are endogenous secreted proteins that reduce endothelial transcytosis of LDL. The first 2 are under investigation for improving cardiac function after myocardial infarction (see the study by Korf-Klingebiel et al^100^ and www.regencor.com). BMP9 circulates at measurable levels and is regarded as a vascular quiescence and stability factor with promising therapeutic potential^34^; it has been used chronically in mice for inhibiting pulmonary arterial hypertension. Downregulating SR-B1 by using G1 to target GPER or by using metformin appears to be worth investigating since the former might affect endothelial cells more than hepatic cells and the latter has a beneficial therapeutic profile in type 2 diabetes.

Despite the current focus on receptors and related pathways, targeting general vesicular machinery by repurposing 2 existing drugs—statins and colchicine—might provide the simplest and safest method for reducing transcytosis. They cost little, have an acceptable safety profile, and can be taken long term. Further work is required to confirm the effects on LDL transcytosis in vivo, but both are already known to have beneficial influences on other cardiovascular risk factors. If lowering cholesterol also lowers endothelial permeability to cholesterol, that might help explain the observation^101^ that risk of coronary heart disease is more than linearly related to serum cholesterol concentration. Ironically, it would also dilute one of the motivations for studying transcytosis. Lowering circulating LDL concentrations and lowering permeability should in combination have a multiplicative effect on subendothelial accumulation.^91^ However, it seems that the primary LDL-lowering therapy used in current clinical practice may already lower permeability.

We conclude by describing some unknowns in the search for potential therapeutic targets.

The first is how vesicles actually transport material across cells. The original concept of Palade^102^ and others was a combination of endocytosis, transport of the vesicle with its contents across the cell, and then exocytosis. However, this ferry-boat model has been questioned. When the 3-dimensional structure of caveolae within endothelial cells was reconstructed from electron microscope images of serial ultrathin sections, almost all the caveolae were found to be connected to the luminal or abluminal plasma membrane or to other caveolae so connected. That was true for capillaries snap-frozen at liquid helium temperatures and embedded by freeze substitution,^103^ as well as for those embedded after chemical fixation,^104–106^ which, it has been argued, could artifactually create such connections. Caveolae that appear to be free in the cytoplasm in single sections are in fact connected to another vesicle or to the plasma membrane out of the plane of the section. A related finding is that caveolae are immobile, albeit in nonendothelial cells.^107^ The near absence of free vesicles and the apparent immobility of caveolae appear to invalidate the concept of vesicles as cargo-carrying ferry boats.

Two other possibilities are shuttling of contents from one vesicle to another by transient fusion^108^ and the formation by vesicle fusion of patent tubes from one side of the cell to the other.^108,109^ One argument for patent tubes is that they would allow advection of LDL and hence explain effects of pressure on its rate of transport. However, pressure might also increase vesicular transport by stretching and flattening the endothelium, thereby increasing area and decreasing path length.

A second unknown is whether receptors are involved in some, most, or all LDL transcytosis. The TIRF microscopy method used in many studies visualizes, by intention, only particles that will bind to the plasma membrane; fluid-phase transcytosis and transport through patent vesicular tubes are excluded. That may have exaggerated the importance of receptors, and the same is true of the concentrations of LDL used in most studies, which are generally around 2 orders of magnitude lower than the Western average plasma concentration of 0.7 mg protein/mL (3.5 mg LDL per mL). The receptors may saturate at well below physiological concentrations and hence only account for a small fraction of the total LDL transport. (LDLR-mediated transcytosis across BBB endothelium, for example, was nearly saturated at 40 µg/mL.^110^)

On the other hand, eliminating endothelial expression of SR-B1 or Alk1 resulted in impressive, 50% reductions in LDL transport in vivo and 70% when both were inhibited,^15^ suggesting that these receptors are of crucial importance, at least in the mouse. Of course, the receptors are likely to increase transcytosis by concentrating LDL within the caveolae. However, additional mechanisms are possible. For example, binding of albumin to Gp60 triggers transcytosis by phosphorylating Src family tyrosine kinases and caveolin-1.^111^ That will increase transport of the bound albumin but presumably also of other plasma solutes that are engulfed at the same time, including LDL. Up to 7 LDL particles could be close-packed within 1 caveola. If the second mechanism also applies to LDL-receptor interactions, then eliminating those receptors will reduce fluid-phase as well as receptor-mediated transcytosis, perhaps accounting for some of the confusion.

A related question is whether transcytosis is an active process. Vasile et al,^7^ who used physiological LDL concentrations and observed a low sensitivity to temperature, thought it was not, but the temperature insensitivity has not been replicated,^112^ and current orthodoxy favors an active process. Active, directional transcytosis would explain why concentrations of free LDL in the subendothelial interstitial fluid are twice those in plasma^113^ and why LDL does not accumulate in the intima where endothelial cells are missing.^7^

A fourth question is how to target only transcytosis that is harmful. It would presumably be beneficial to reduce LDL transcytosis into the artery wall, but not LDL transport out, not HDL transport in or out, and not receptor-mediated handling of lipoproteins in the liver. Furthermore, inward LDL transport would ideally be reduced only in regions of arteries that are prone to atherosclerosis. That might be achieved by adding endogenous compounds (such as FSTL1) that are expressed at insufficient levels only under proatherogenic flow conditions or by targeting therapeutics to endothelium expressing inflammatory markers; VCAM-1 (vascular cell adhesion protein 1)–targeting nanoparticles that interfere with MFSD2a signaling have been used to weaken the blood-tumor barrier and hence improve delivery of chemotherapeutics.^114^

## ARTICLE INFORMATION

### Sources of Funding

I.O. Bolanle was supported by project grant PG/23/11466 from the British Heart Foundation.

### Disclosures

None.
